# Left sided breast cancer is associated with aggressive biology and worse outcomes than right sided breast cancer

**DOI:** 10.1038/s41598-022-16749-4

**Published:** 2022-08-04

**Authors:** Yara Abdou, Medhavi Gupta, Mariko Asaoka, Kristopher Attwood, Opyrchal Mateusz, Shipra Gandhi, Kazuaki Takabe

**Affiliations:** 1 Department of Medical Oncology, University of North Carolina, Chapel Hill, NC 27514 USA; 2 Warren Alpert School of Medicine, Brown University, Women & Infants Hospital, Providence, RI 14203 USA; 3 Department of Surgical Oncology, Roswell Park Comprehensive Cancer Center, Buffalo, NY 14203 USA; 4 Division of Medical Oncology, Department of Statistics, Roswell Park Comprehensive Cancer Center, Buffalo, NY 14203 USA; 5 Division of Medical Oncology, Washington University School of Medicine, Saint Louis, MO 63110 USA; 6 Division of Medical Oncology, Roswell Park Comprehensive Cancer Center, Buffalo, NY 14203 USA; 7 Department of Surgery, University at Buffalo Jacobs School of Medicine and Biomedical Sciences, The State University of New York, Buffalo, NY USA; 8 Department of Surgery, Niigata University Graduate School of Medical and Dental Sciences, Niigata, 951-8510 Japan; 9 Department of Surgery, Yokohama City University, Yokohama, 236-0004 Japan; 10 Department of Breast Surgery and Oncology, Tokyo Medical University, Tokyo, 160-8402 Japan; 11 Department of Breast Surgery, Fukushima Medical University School of Medicine, Fukushima, 960-1295 Japan

**Keywords:** Breast cancer, Cancer genomics

## Abstract

Breast cancer is more common on the left side than the right side. We aim to evaluate differences in clinicopathological and genomic characteristics based on laterality. We analyzed survival outcomes and clinical characteristics of 881,320 patients recorded by the Surveillance, Epidemiology, and End Results (SEER) program. The Cancer Genome Atlas (TCGA) was used to explore genomic and clinical features from 1,062 patients. Gene expression data was used to quantitate cytolytic activity and hallmark gene-sets were used for gene set enrichment analysis. An institutional retrospective review was conducted on 155 patients treated with neoadjuvant chemotherapy (NACT). Patient characteristics were summarized by pathological complete response (pCR). Left sided tumors were found to be more prevalent than right sided tumors. No major clinicopathological differences were noted by laterality. Left sided breast cancer demonstrated poorer outcomes versus right sided tumors (HR 1.05, 95% CI 1.01–1.08; p = 0.011). Cell proliferation gene sets, including E2F Targets, G2M Checkpoint, Mitotic spindle, and MYC Targets, were enriched on the left side compared to the right. Left sided tumors had lower pCR rates versus right sided tumors (15.4% versus 29.9%, p = 0.036). Our findings suggest that left sided breast cancer is associated with aggressive biology and worse outcomes compared to right sided breast cancer.

## Introduction

Published literature on breast cancer laterality is limited, with the first study published approximately 80 years ago by Fellenberg et al.^1^ raising awareness to an increased frequency of left sided breast cancer. Since then, it has been consistently reported that women are slightly more likely to be diagnosed with breast cancer in the left breast than in the right, with the ratio of left to right side tumors ranging between 1.05 to 1.26^2–4^. Despite the consistency of this predominance of left sided breast cancer, meaningful underlying characteristic differences between the two sides have not been well investigated.

The breast is a paired organ and both sides share identical genetic and environmental risk factors that contribute to cancer development. However, studies have shown that paired organs may differ in their tissue structure, arterial and venous supplies and lymphatic drainage during embryonic development^5^ leading to biological differences between both sides and a possible association with cancer laterality^6^.

Prior studies have tried to explain this left sided predominance through various hypothesis including, larger size of left breast^7^, early detection of left tumors in right handed women^8^, and preferential right sided breast feeding^9^. However, these explanations have not been confirmed by subsequent research or uniformly accepted. Various factors on breast cancer laterality have been reported such as; laterality ratio varies with age^4,10–12^, different quadrants of the breast may have different laterality ratios^3,11^, and possible correlation between laterality and genetic inheritance^2^. Nevertheless, this subject continues to be a complex and poorly understood phenomenon.

Recently our group has been investigating the cancer biology of breast cancer using clinical and gene expression data from The Cancer Genome Atlas (TCGA)^13–19^. Computational algorithms such as cytolytic activity score (CYT)^17,18,20–23^ and gene set enrichment analysis (GSEA)^17–19,23^ allow us to characterize the biological quality of the tumor.

Here we hypothesize that left-sided breast cancer is biologically aggressive and has worse outcomes compared to right-sided breast cancer. We evaluate the clinical and pathological differences between left and right sided breast cancer using a large patient cohort. We also evaluate differences in cancer biology by computational biological analyses. Finally, we investigate the clinical relevance of laterality by analyzing a neoadjuvant cohort.

## Methods

### The surveillance, epidemiology, and end results (SEER) database analyses

SEER database was interrogated for n = 1,614,013 breast cancer patients with laterality information from 1988 to 2015. Male patients, patients < 18 years of age, and patients with bilateral disease, DCIS, stage IV, missing stage or survival outcomes were excluded from analysis. Patient demographic and clinical characteristics were summarized by side (left versus right) using the appropriate descriptive statistics, with comparisons made using the Mann–Whitney U or Pearson chi-square tests. Overall Survival (OS) and Cancer Specific Survival (CSS) were summarized by side (in the overall sample and within each AJCC stage) using standard Kaplan–Meier methods, where estimates of the median OS and CSS were obtained with 95% confidence intervals (CIs). Comparisons were made using the log-rank test. Multivariable analyses were conducted using Cox regression models, where the demographic and clinical characteristics in Table 1 were included as covariates (selected a priori). Hazard ratios (HRs) and corresponding 95% confidence intervals were obtained from model estimates. To evaluate the impact of side within demographic and clinical subgroups, Cox regression models were fit in a one-at-a-time manner with side, the sub-group variable, and their two-way interaction as predictors. From the model estimates, hazard ratios are obtained for side within each sub-group and Wald chi-square tests were used to evaluate the interaction term. Analyses were conducted in SAS v9.4 (Cary, NC) at a significance level of 0.05. SEER database is a deidentified public database, therefore no institutional review board (IRB) approval was needed.

### Computational biology analyses

Clinical and gene expression data in n = 1,062 breast cancer patients (552 left sided, 510 right sided) were obtained from The Cancer Genome Atlas (TCGA) as we have previously described^13–19^. Lymphovascular Invasion (LVI) status, mitotic rate, nuclear score and tubular score and Nottingham pathological grade score data were collected manually from pathology reports in TIES client 5.8. Fisher's exact test was used for group comparisons. Cytolytic activity (CYT) indicates anti-cancer immune response and was quantified from gene expression data as we have previously described^17,18,20–25^. Hallmark gene-sets were used for gene set enrichment analysis (GSEA) as previously described^17–19,23,26,27^.

### Analyses of a neoadjuvant chemotherapy cohort

A retrospective, single-institute review was conducted for a total of n = 155 breast cancer patients treated at Roswell Park Comprehensive Cancer Center, Buffalo NY from 2009 to 2013. Eligible patients were treated with neoadjuvant chemotherapy, followed by either a lumpectomy or mastectomy. Pathologic complete response (pCR) was defined as an absence of any evidence of invasive carcinoma in the surgical pathology specimen i.e., ypT0/is N0. Patient characteristics were summarized by pCR using the appropriate descriptive statistics; with comparisons were made using the Mann–Whitney U or Fisher’s exact tests. A multivariate logistic regression model was used to evaluate the association between side and pCR while adjusting for disease grade and hormone receptor status. Odds ratios, with 95% CIs, were obtained from model estimates. Analyses were conducted in SAS v9.4 (Cary, NC) at a significance level of 0.05. The research study was performed in accordance with the Declaration of Helsinki. The study was approved by the local IRB and the requirement for informed consent was waived because of the retrospective nature of this study.

## Results

### Clinicopathological features of left and right sided breast cancer

In the SEER database, 881,320 patients were included in this analysis. Left sided and right sided tumors were seen in 50.8% and 49.2% of patients respectively. The baseline demographic and clinical characteristics based on laterality are summarized in Table 1. Poorly differentiated and undifferentiated tumors, as well as hormone negative and HER2 positive tumors were significantly more prominent on the left side compared to the right side (p < 0.001). In the TCGA cohort, left sided and right sided tumors were seen in 52.0% and 48.0% of patients respectively. A higher number of grade 2 and 3 tumors was seen on the left side compared to the right (p = 0.02). No other statistically significant differences in clinical features were seen in that cohort (Table 2). Lastly, in our institutional cohort of 155 patients, left sided and right sided tumors were seen in 50.3% and 49.7% of patients respectively. No statistically significant differences were seen in clinicopathological characteristics (Supplemental Table S1).

**Table 1:** The Surveillance, Epidemiology, and End Results (SEER) program: baseline characteristics based on laterality.

	Left	Right	Overall	P-value
**Overall**				
N (%)	447,401 (50.8%)	433,919 (49.2%)	881,320 (100%)	
**Age**				
< 45	59,138 (13.2%)	58,582 (13.5%)	117,720 (13.4%)	< 0.001
45–65	214,751 (48.0%)	209,059 (48.2%)	423,810 (48.1%)	
65 +	173,512 (38.8%)	166,278 (38.3%)	339,790 (38.6%)	
**Grade**				
Well diff	84,957 (21.0%)	84,542 (21.5%)	169,499 (21.2%)	< 0.001
Moderately diff	172,823 (42.6%)	168,392 (42.8%)	341,215 (42.7%)	
Poorly diff	141,671 (35.0%)	135,057 (34.3%)	276,728 (34.6%)	
Undifferentiated	5,880 (1.5%)	5,580 (1.4%)	11,460 (1.4%)	
**AJCC stage**				
I	221,671 (49.5%)	215,580 (49.7%)	437,251 (49.6%)	0.444
II	163,139 (36.5%)	157,805 (36.4%)	320,944 (36.4%)	
III	62,591 (14.0%)	60,534 (14.0%)	123,125 (14.0%)	
**ER**				
Negative	82,598 (20.6%)	77,862 (20.0%)	160,460 (20.3%)	< 0.001
Positive	318,300 (79.4%)	311,694 (80.0%)	629,994 (79.7%)	
**PR**				
Negative	124,578 (31.5%)	117,880 (30.7%)	242,458 (31.1%)	< 0.001
Positive	271,391 (68.5%)	266,691 (69.3%)	538,082 (68.9%)	
**HER2**				
Negative	117,577 (85.1%)	114,648 (85.4%)	232,225 (85.2%)	0.041
Positive	20,597 (14.9%)	19,645 (14.6%)	40,242 (14.8%)	
**Hormone receptor**				
Triple negative	15,818 (11.5%)	14,976 (11.2%)	30,794 (11.3%)	< 0.001
Triple positive	10,195 (7.4%)	9,796 (7.3%)	19,991 (7.4%)	
ER + /PR + /HER2-	88,385 (64.2%)	87,035 (65.0%)	175,420 (64.6%)	
ER-/PR-/HER2 +	6,199 (4.5%)	5,841 (4.4%)	12,040 (4.4%)	
ER or PR +	17,065 (12.4%)	16,184 (12.1%)	33,249 (12.2%)	
**Nodes positive**				
0	273,666 (67.1%)	265,830 (67.1%)	539,496 (67.1%)	< 0.001
1–3	90,701 (22.2%)	87,494 (22.1%)	178,195 (22.2%)	
4–9	29,646 (7.3%)	28,218 (7.1%)	57,864 (7.2%)	
10 +	13,984 (3.4%)	14,360 (3.6%)	28,344 (3.5%)	

*ER* estrogen receptor, *PR* progesterone receptor, *HER2* human epidermal growth factor receptor 2, *AJCC* American Joint Committee on Cancer, *Min* minimum, *Max* maximum.

**Table 2:** The Cancer Genome Atlas (TCGA): baseline characteristics based on laterality.

Variable	Left	Right	P-value
**Overall**			
N (%)	552 (52%)	510 (48%)	
**Grade**			
1	28 (5.0%)	48 (9.2%)	0.021
2	144 (25.7%)	122 (23.5%)	
3	121 (21.6%)	105 (20.2%)	
**Stage**			
I	92 (16.4%)	90 (17.3%)	0.76
II	324 (57.8%)	287 (55.2%)	
III	125 (22.3%)	121 (23.3%)	
**Tumor size**			
T1	139 (24.8%)	139 (26.7%)	0.485
T2	324 (57.8%)	292 (56.2%)	
T3	67 (11.9%)	66 (12.7%)	
T4	21 (3.7%)	12 (2.3%)	
**Nodal involvement**			
Negative	255 (45.5%)	225 (43.3%)	0.537
Positive	296 (52.8%)	284 (54.6%)	
**Subtype**			
ER+/PR+/HER2−	205 (36.5%)	182 (35.0%)	0.766
ER+/PR+/HER2+	65 (11.6%)	53 (10.2%)	
ER−/PR−/HER2+	23 (4.1%)	16 (3.1%)	
Triple negative	56 (10.0%)	56 (10.8%)	
**ER status**			
Negative	125 (22.3%)	107 (20.6%)	0.502
Positive	400 (71.3%)	379 (72.9%)	
**PR status**			
Negative	175 (31.2%)	155 (29.8%)	0.591
Positive	346 (61.7%)	331 (63.7%)	
**Her2 status**			
Negative	287 (51.2%)	260 (50.0%)	0.53
Positive	88 (15.7%)	70 (13.5%)	

*ER* estrogen receptor, *PR* progesterone receptor, *Her-2* human epidermal growth factor receptor 2.

### Survival outcomes of left and right sided breast cancer

**Figure 1:**
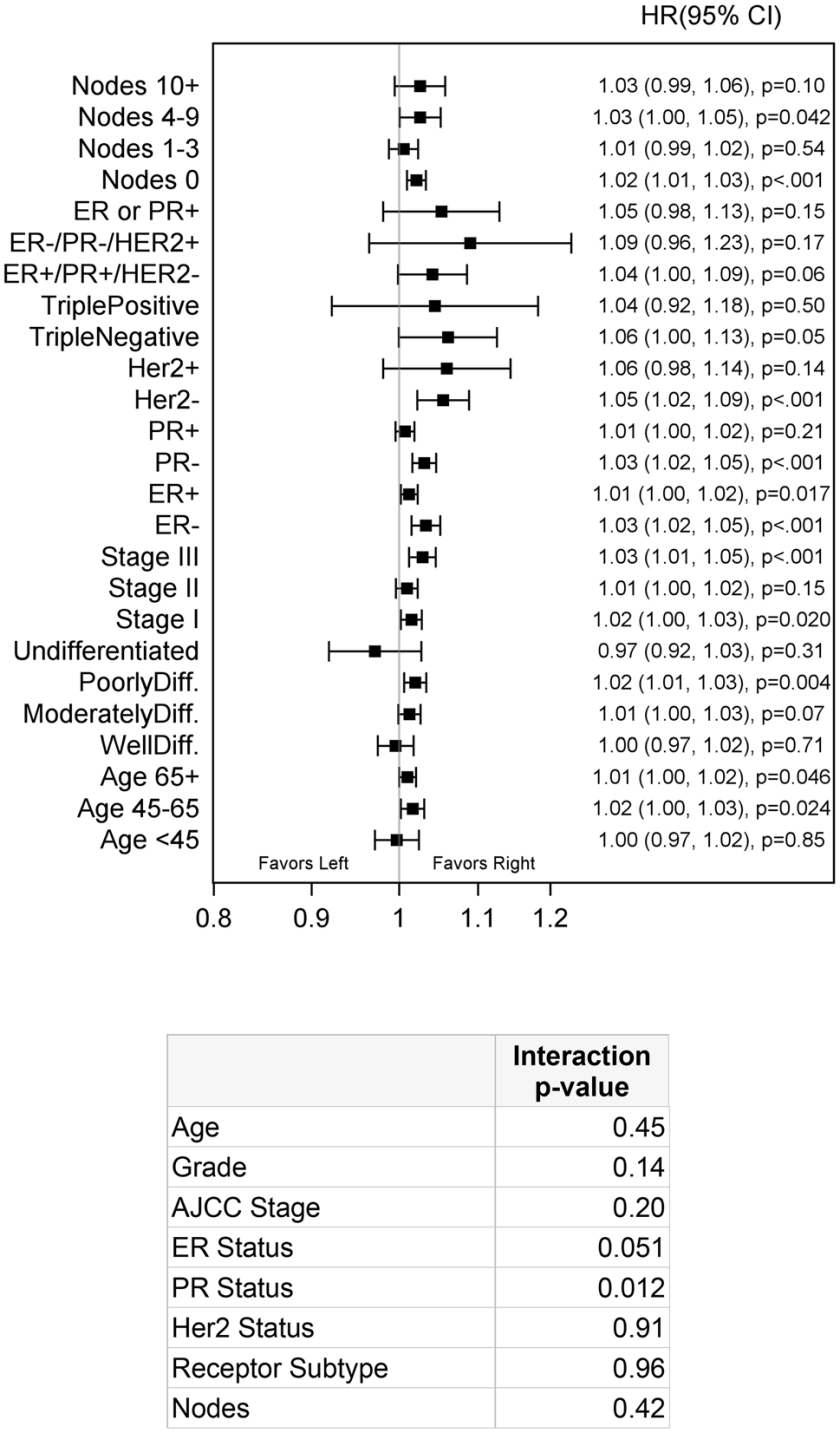
The Surveillance, Epidemiology, and End Results (SEER) program: interaction analysis. *ER* estrogen receptor, *PR* progesterone receptor, *HER2* human epidermal growth factor receptor 2, *AJCC* American Joint Committee on Cancer, *Diff* differentiated.

In the SEER population, left sided breast cancer demonstrated poorer OS and CSS compared to right sided breast cancer (left vs right HR 1.02, p < 0.001, Table 3). Left sided tumors continued to show poorer outcomes even after adjusting for grade, stage, and hormone receptor status (left vs right HR 1.05, 95% CI 1.01,1.08; p = 0.011; Table 4). The results of the interaction analysis showed no significant interaction between laterality and age, grade, stage, receptor status or number of positive nodes (Fig. 1). Although tumors that are ER and/or PR negative appear to have worse outcomes on the left side (HR 1.03, p < 0.001). No significant differences in survival were noted in the TCGA or institutional cohorts (data not shown).

**Table 3:** Overall survival and cancer-specific survival rates in left and right breast cancer.

	Overall survival				Cancer specific survival			
	5 yr rate (95% CI)	10 yr rate (95% CI)	Hazard ratio (95% CI)	P-value	5 yr rate (95% CI)	10 yr rate (95% CI)	Hazard ratio (95% CI)	P-value
Total	84.7 (84.6, 84.8)	70.6 (70.5, 70.7)	–	< 0.001	91.4 (91.4, 91.5)	85.4 (85.3, 85.5)	–	< 0.001
Left	84.5 (84.4, 84.6)	70.4 (70.3, 70.6)	1.02 (1.01, 1.03)		91.3 (91.2, 91.4)	85.3 (85.1, 85.4)	1.02 (0.01, 1.04)	
Right	84.9 (84.8, 85.0)	70.8 (70.6, 71.0)	1.00		91.6 (91.5, 91.7)	85.6 (85.5, 85.7)	1.00	

*Yr* year, *CI* confidence interval.

**Table 4:** Multivariate survival analysis while adjusting for grade, stage and tumor subtype.

Variable	Hazard ratio	P-value
**Laterality**		
Left vs right	1.05 (1.01, 1.08)	0.011
**Age**		
45–65 vs < 45	1.05 (0.99, 1.12)	< 0.001
65 + vs < 45	2.92 (2.74, 3.10)	
**Grade**		
Moderately diff. vs well diff	1.12 (1.05, 1.18)	< 0.001
Poorly diff. vs well diff	1.68 (1.59, 1.79)	
Undifferentiated vs well diff	1.98 (1.59, 2.48)	
**AJCC stage**		
II vs I	1.71 (1.62, 1.80)	< 0.001
III vs I	3.80 (3.52, 4.10)	
**Nodes positive**		
1–3 vs 0	1.28 (1.22, 1.34)	< 0.001
4–9 vs 0	1.30 (1.20, 1.41)	
10 + vs 0	2.08 (1.91, 2.26)	
**Hormone receptor**		
Triple positive vs Triple negative	0.37 (0.34, 0.40)	< 0.001
ER+/PR+/HER2− vs triple negative	0.40 (0.38, 0.42)	
ER−/PR−/HER2+ vs Triple negative	0.54 (0.50, 0.58)	
ER/PR+ vs triple negative	0.64 (0.60, 0.67)	

*Yr* year, *CI* confidence interval, *ER* estrogen receptor, *PR* progesterone receptor, *HER2* human epidermal growth factor receptor 2, *AJCC* American Joint Committee on Cancer.

### Transcriptome profiles and biological characteristics of left and right sided breast cancer

**Figure 2:**
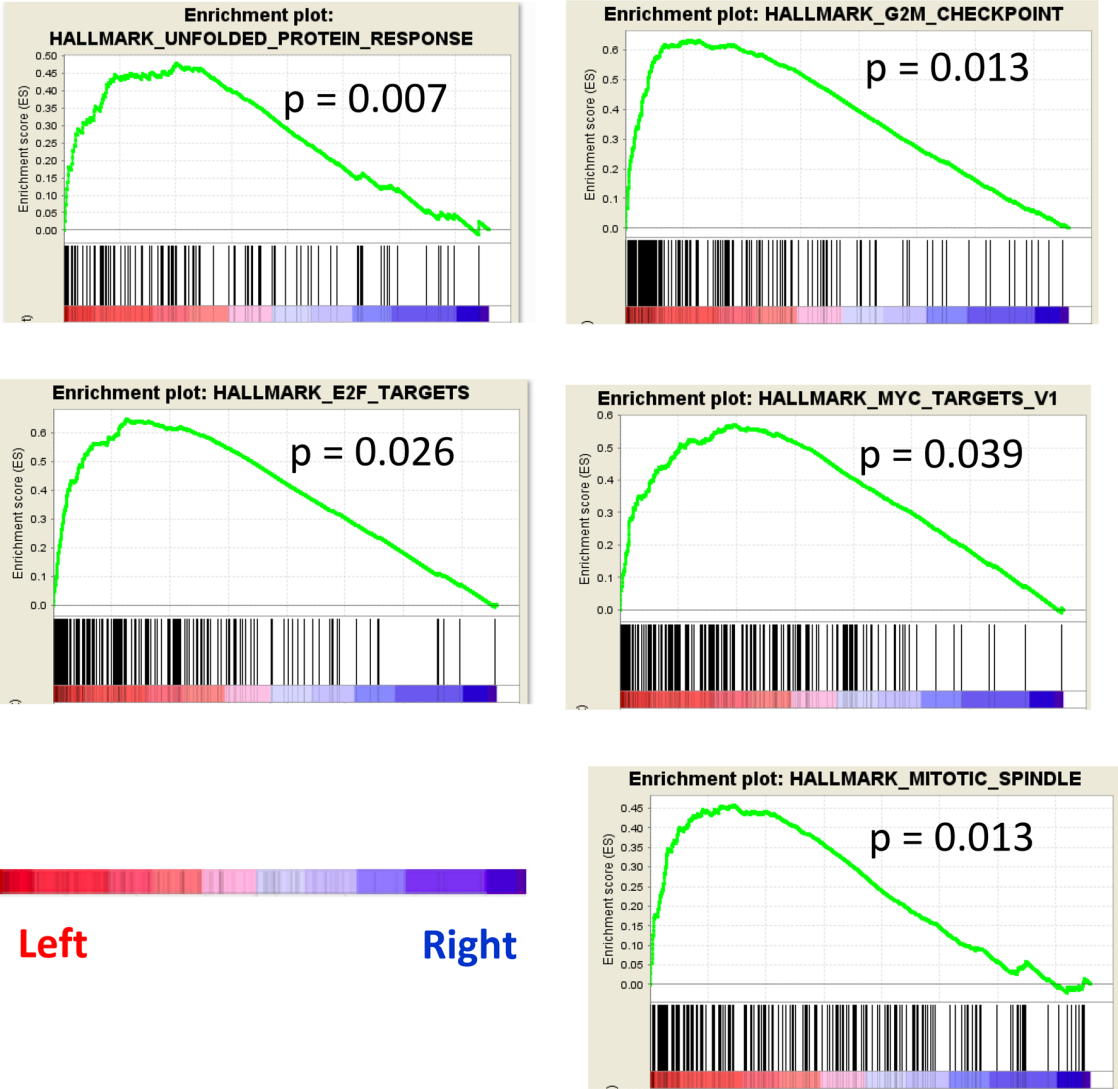
Proliferation related gene sets enriched in Left breast cancer.

GSEA demonstrated that cell proliferation and cell-cycle related gene sets including G2M checkpoint, Mitotic spindle, E2F targets and MYC targets, were significantly enriched in left sided tumors (Fig. 2). Furthermore, out of the 865 genes that were significantly highly expressed on the left side, we identified specific genes that are associated with breast tumorigenesis: including BRCA1, BRCA2, BRIP1, CHEK2, FANCC, PALB2, TP53 and MSH6. No statistically significant differences were observed in mutation count and CYT between the sides (Supplemental Fig. S1).

### Pathological complete response in left and right sided breast cancer

**Figure 3:**
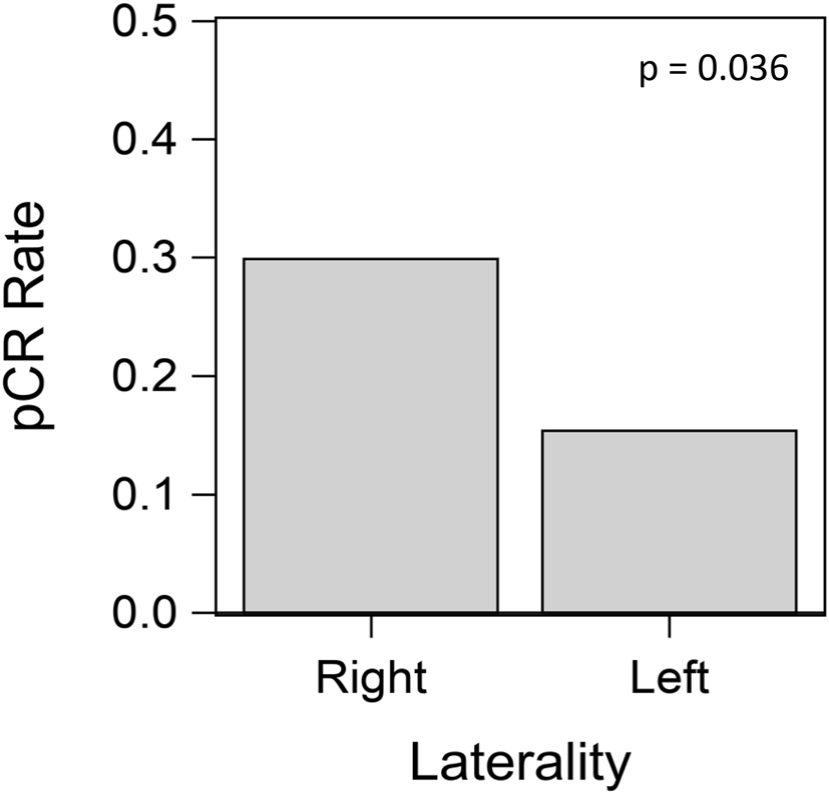
Pathological Complete Response (pCR) in left and right sided breast cancer.

In our institutional cohort of 155 patients that underwent neoadjuvant chemotherapy, there was a lower rate of pathologic complete response (pCR) in response to neoadjuvant chemotherapy with left sided tumors as compared to right sided tumors (15.4% versus 29.9% respectively, p = 0.036) (Fig. 3). This held true when adjusted for grade, stage, and receptor status (OR 0.43 (0.18–0.99), p = 0.048).

## Discussion

The most recently published comprehensive analysis of breast cancer laterality in a large population cohort (SEER), analyzed patients between 1973 and 2010^11^. Our updated SEER analysis confirms the persistence of left breast cancer predominance; with a slightly higher prevalence of ER/PR negative and HER-2 positive tumors on the left. The significant differences seen in clinicopathological variables by laterality appear to have no clinical relevance and are largely driven by the large sample sizes. However, we do interestingly note a slight survival disadvantage to left sided breast cancer compared to right side with a hazard ratio of 1.05. In another SEER analysis, Bao et al. showed slightly worse breast cancer specific mortality in the left central portion of the breast (HR, 1.100; P = 0.013, using the right side as the reference), with no other significant interactions between laterality and primary tumor site seen in their study^28^. Our study did not include specific information on primary tumor site within each breast, therefore, we are not able to make definitive conclusions regarding the relationship between laterality and the tumor location within each breast. Most other previous studies showed no difference in survival outcomes by breast cancer laterality^2,3,5,10,29^, although, there was a single study of 5,459 breast cancer patients in Egypt which showed significantly worse survival in left sided breast cancer compared to right^30^. That being said, patients with left sided breast cancer treated with radiation therapy were at greater risk of cardiovascular complications, and historically had worse outcomes^31,32^.

While many studies discussed possible causes for left sided predominance in breast cancer^7–9,12,33,34^, no previous research have looked at underlying genomic differences between left and right sided breast cancer. We noted no differences in tumor infiltrating immune cells by laterality, however, several cell proliferation gene sets were found to be significantly enriched on the left side, indicating distinct underlying biologic characteristics between the two sides. This observation was further illustrated in our institutional analysis of the neoadjuvant cohort, where left sided tumors were less likely to achieve a pCR compared to right sided tumors. Even though prior research considered higher proliferation rates to be a predictor of pCR^35^, left sided tumors had lower rates of pCR in our institutional cohort, despite having more prominent cell proliferation gene sets on that side. These findings suggest the presence of a complex interplay between the biology of left sided tumors and response to treatment, regardless of other clinical characteristics. Additionally, more comprehensive approaches, such as investigating the role of the tumor microenvironment and comparing differences in immune cells by breast cancer laterality, may provide further insight to this hypothesis. Even among the cancer cells, it may be interesting to investigate the differences in intertumoral heterogeneity by laterality. In conclusion, we cannot help but speculate whether left sided breast cancer is more prominent than right sided breast cancer due to an underlying biological process that was not previously discussed in literature, and whether this distinct process makes left sided breast cancer more chemo-resistant than right sided cancer given the noted lower pCR rates. That being said, the close similarity in survival outcomes between both sides may stir us away from taking a more aggressive management approach with left sided tumors.

This study has certain limitations. Being a retrospective analysis, we are unable to determine definitive causation to the excess number of tumors found in the left breast but could only show association. Furthermore, possible confounding variables could not be identified retrospectively, due to lack of awareness regarding how they interact with our outcomes and results, although multivariate analysis was used to control for confounding variables. Another limitation is the large number of missing HER2-expression data in our cohort. Therefore, one can argue that receptor status can be a surrogate for the gene expression subtype classification reported by Perou et al.^36^ in only a minority of the cohort. Although, many clinicians make clinical decisions using the receptor status, and not the surrogate of the molecular subtype. To this end, we believe our results on receptor status are informative to such clinicians. Despite these limitations, our study provides novel data on breast cancer laterality and adds a unique perspective to underlying biology of left sided breast cancer.

## Conclusions

In conclusion, we observe that left sided breast tumors have a more proliferative genomic profile, lower responses to neoadjuvant chemotherapy, and slightly worse long-term outcomes compared to right sided breast cancer. These findings are quite novel and if validated, may enhance our management approaches in the future.

## Supplementary Information

Supplementary Information.

## Data Availability

The datasets used and/or analyzed during the current study are available from the corresponding author on reasonable request.
