# Supplementary material for: Left sided breast cancer is associated with aggressive biology and worse outcomes than right sided breast cancer
Source: Sci Rep. 2022 Aug 4;12:13377. doi: 10.1038/s41598-022-16749-4 (PMC9352772; doi:10.1038/s41598-022-16749-4)
Supplement: Supplementary file 1 — Supplementary Information. [file 41598_2022_16749_MOESM1_ESM.doc]

Supplementary Appendix

**Supplemental Table 1. Single Institution Analysis: Baseline Characteristics Based on Laterality**

|  | | **Right** | **Left** | **P-value** |
| --- | --- | --- | --- | --- |
| Overall | N | 77 (49.7%) | 78 (50.3%) | 0.939 |
| Age | Median/Min/Max | 52.0/23.0/85.0 | 52.5/27.0/80.0 |  |
| Body mass index | Mean/Std/N | 28.8/7.2/77 | 57.5/236.9/77 | 0.089 |
|  | Median/Min/Max | 27.0/17.1/49.3 | 29.7/18.1/2108.0 |  |
| Histology | Ductal | 69 (89.6%) | 65 (83.3%) | 0.678 |
|  | Lobular | 3 (3.9%) | 6 (7.7%) |  |
|  | Ductal & Lobular | 1 (1.3%) | 2 (2.6%) |  |
|  | Other | 4 (5.2%) | 5 (6.4%) |  |
| Grade | 1 | 6 (8.0%) | 11 (14.1%) | 0.448 |
|  | 2 | 23 (30.7%) | 20 (25.6%) |  |
|  | 3 | 46 (61.3%) | 47 (60.3%) |  |
| T-stage | T1 | 6 (7.8%) | 8 (10.3%) | 0.512 |
|  | T2 | 34 (44.2%) | 35 (44.9%) |  |
|  | T3 | 23 (29.9%) | 16 (20.5%) |  |
|  | T4 | 14 (18.2%) | 19 (24.4%) |  |
| N-stage | N0 | 28 (36.4%) | 32 (41.0%) | 0.622 |
|  | N+ | 49 (63.6%) | 46 (59.0%) |  |
| M-stage | M0 | 77 (100.0%) | 76 (97.4%) | 0.497 |
|  | M+ |  | 2 (2.6%) |  |
| Receptor Status | HR+/HER2- | 31 (40.3%) | 33 (42.9%) | 0.621 |
|  | HR+/HER2+ | 15 (19.5%) | 9 (11.7%) |  |
|  | HR-/HER2+ | 8 (10.4%) | 9 (11.7%) |  |
|  | Triple Negative | 23 (29.9%) | 26 (33.8%) |  |
| Treatment | AC= No | 18 (23.7%) | 12 (15.6%) | 0.228 |
|  | AC= Yes | 58 (76.3%) | 65 (84.4%) |  |
|  | T= No | 1 (1.3%) | 3 (3.9%) | 0.620 |
|  | T= Yes | 75 (98.7%) | 74 (96.1%) |  |
| Pathologic complete response (pCR) | Residual disease | 54 (70.1%) | 66 (84.6%) | 0.036 |
|  | Complete response | 23 (29.9%) | 12 (15.4%) |  |

Abbreviations: HR=Hormone Receptor (Estrogen Receptor or Progesterone Receptor), HER2=Human Epidermal Growth Factor Receptor 2, Min= Minimum, Max=Maximum, Std= Standard, AC= Adriamycin + Cyclophosphamide, T= Taxane

**Supplemental Figure 1****. Mutation count and CYT analysis between Left and Right breast cancer**
